# The Relationship between Selective Digestive Decontamination and Nosocomial Infections in Patients Receiving Continuous Renal Replacement Therapy in ICUs: A Multicenter Study

**DOI:** 10.3390/jcm13144211

**Published:** 2024-07-19

**Authors:** Juan Luis Vicente Arranz, Catalina Sánchez-Ramírez, Pedro Saavedra, Yasmina Rivero Perdomo, María Victoria Lorenzo-Martín, José Blanco-López, Casimira Domínguez Cabrera, Carmen-Rosa Hernández-Socorro, Sergio Ruiz-Santana

**Affiliations:** 1 Intensive Care Unit, Hospital Universitario de Gran Canaria Dr. Negrín, University of Las Palmas de Gran Canaria, Barranco de la Ballena s/n, E-35010 Las Palmas de Gran Canaria, Spain; jvicarr@gobiernodecanarias.org (J.L.V.A.); csanrams@gobiernodecanarias.org (C.S.-R.); yrivper@gobiernodecanarias.org (Y.R.P.); 2 Department of Mathematics, University of Las Palmas de Gran Canaria, E-35010 Las Palmas de Gran Canaria, Spain; pedro.saavedra@ulpgc.es; 3 Intensive Care Unit, Complejo Hospitalario Universitario Insular-Materno Infantil, Avenida Marítima del Sur s/n, E-35016 Las Palmas de Gran Canaria, Spain; mlormar@gobiernodecanarias.org (M.V.L.-M.); jblalop@gobiernodecanarias.org (J.B.-L.); 4 Central Laboratory, Department of Clinical Analysis, Hospital Universitario de Gran Canaria Dr. Negrín, University of Las Palmas de Gran Canaria, Barranco de la Ballena s/n, E-35010 Las Palmas de Gran Canaria, Spain; cdomcab@gobiernodecanarias.org; 5 Department of Radiology, Hospital Universitario de Gran Canaria Dr. Negrín, University of Las Palmas de Gran Canaria, Barranco de la Ballena s/n, E-35010 Las Palmas de Gran Canaria, Spain; carmenrosa.hernandez@ulpgc.es

**Keywords:** selective digestive decontamination, nosocomial infections, renal failure, renal replacement therapy

## Abstract

**Background:** Nosocomial infections are a worldwide healthcare issue, especially in intensive care units (ICUs), and they had a prevalence of 21.1% in 2023 in Spain. Numerous predisposing risk factors have been identified, with the most relevant being invasive techniques, including renal replacement therapies (RRTs). Several outstanding strategies have been published that prevent or reduce their incidence, including the nationwide ZERO in Spain, which consists of structured guidelines to be implemented to tackle this problem. One of these strategies, which is defined as ‘highly recommended’ in these projects, is selective digestive decontamination (SDD). The main aim of this study is to compare the incidences of ICU-acquired infections, including those due to multidrug-resistant bacteria (MDRB), in two cohorts of RRT with or without SDD. **Methods:** We conducted a multicenter, prospective, observational study at two tertiary hospitals in Spain. In total, 140 patients treated with RRT were recruited based on their exposure to SDD. Surveillance microbiological samples and nosocomial infection risk factors were obtained. Infection rates per 1000 days of exposure and the MDRB incidence density ratio were determined. **Results:** SDD statistically significantly reduced RRT-associated nosocomial infections (OR: 0.10, 95% CI: (0.04–0.26)) and the MDRB incidence density ratio (IDR: 0.156, 95% CI = 0.048–0.506). However, mechanical ventilation (OR: 7.91, 95% CI: (2.54–24.66)) and peripheral vascular disease (OR: 3.17, 95% CI: (1.33–7.56)) were significantly associated with increases in infections. **Conclusions:** Our results favor the use of SDD in ICU patients with renal failure undergoing CRRT as a tool for infection control.

## 1. Introduction

Renal replacement therapy (RRT) is a technique frequently used in intensive care units (ICUs), with an incidence of 8–10% in critically ill patients [1]. Its main indication has always been acute kidney injury (AKI) [2]. It is estimated that 20% of critical patients with kidney injuries require dialysis in their first week after admission [3]. There are different types of RRT, with continuous renal replacement therapy (CRRT) being by far the most widely used in ICUs. However, like any invasive technique, this therapy has been shown to be a risk factor for the development of nosocomial infections [4,5], with primary and secondary bacteremia, ventilator-associated pneumonia (VAP), and urinary tract infections (UTIs) standing out. AKI “per se”, in addition to being a risk factor for the development of infections, has also been shown to increase the time patients spend in ICUs and short- and long-term mortality [6,7,8,9]. The published data on nosocomial infections in RRT, to our knowledge, are scarce and show that 20% of RRT patients had nosocomial pneumonia among all infected patients recruited [10], or a rate of 5.4 bacteremias per 1000 days of RRT in ICU patients [11]. 

Among the nosocomial infections, those caused by multidrug-resistant bacteria (MDRB) constitute a serious global health problem [12]. Their incidence is increasing, and they are responsible for an increase in mortality. Selective digestive decontamination (SDD) is a therapy that may reduce the incidence of these microorganisms and the infections caused by them [13]. SDD combines the use of non-absorbable topical antibiotics applied to the oropharynx (usually tobramycin or gentamicin, colistin, and nystatin) and gastrointestinal tract with intravenous antibiotics, usually second- or third-generation antibiotics (cefotaxime in our ICU), or a third-generation quinolone in the case of allergy to beta-lactams [13]. In SDD, due to their pharmacokinetics, aminoglycosides usually do not present significant intestinal absorption, making them ideal to act locally in the digestive tract [14]. However, in critically ill patients, the intestinal mucosa may be compromised, facilitating undesired absorption and increasing the risk of secondary toxicity [15]. Therefore, it would be worthwhile to determine the blood levels of these antibiotics, and dose adjustments may be considered in selected patients, as has been demonstrated in several observational studies [14,16]. 

The use of SDD has significantly reduced the incidences of colonization and nosocomial infections, including candidemia and infections caused by MDRB, even in ICUs with greater endemic bacterial resistance [13,17,18,19,20,21]. In this specific setting, SDD does not have clinically relevant impacts on the emergence and spread of resistance or overall systemic antimicrobial use [13,21].

According to the Australian SuDDiCU study, in critically ill patients receiving mechanical ventilation, SDD, compared to standard care without SDD, does not significantly reduce in-hospital mortality but may produce clinically important benefits [22]. Furthermore, this measure has also been suggested to reduce overall mortality [23], as shown by a recently published meta-analysis of 32 clinical trials [24]. The benefits of SDD were evident in trials with an intravenous agent together with the gastrointestinal part of the therapy but were not evident in trials without this agent. SDD has also been associated with reduced risks of ventilator-associated pneumonia and ICU-acquired bacteremia [22,24].

Our intensive care unit has used an SDD regimen with an intravenous agent since 2011. It is applied to patients for whom orotracheal intubation is expected for more than 48 h, as well as patients with a decreased level of consciousness, burns, neutropenia, or previous colonization by an MDRB [13]. In the medical literature, little attention has been paid to the effect of this preventive measure in reducing the rates of nosocomial infections in patients exposed to specific risk factors, such as AKI requiring RRT. Therefore, our main hypothesis was that the incidence of ICU-acquired infections would be lower in an environment where SDD was applied, despite exposure to a well-known risk factor such as renal replacement therapy.

The main aim of this study is to compare the incidences of ICU-acquired infections, including MDRB, in two cohorts of RRT with or without SDD. Our secondary endpoints included identifying preventable risk factors for the development of nosocomial infections in patients treated with RRT and defining whether those infections were mainly caused by MDRB.

## 2. Results

Table 1 summarizes the patient characteristics of the entire cohort and the SDD group. There were no statistically significant differences between the study groups in terms of age, sex, or body mass index (BMI), although the patients in both cohorts were overweight. Almost half of the cohort was diabetic (61, 43.5%), with no statistically significant differences between the groups. The APACHE-II and SOFA scores were shown to be statistically significantly higher in the SDD group compared to the non-SDD group (*p* < 0.001). We can also see that the number of infections during CRRT was significantly lower in the SDD group (*p* < 0.001). We found a significantly higher number of patients with hypertension and heart failure in the SDD group compared to the non-SDD group. Likewise, patients had significantly more admissions due to cardiac surgery and cardiogenic shock in the group receiving SDD (*p* < 0.05), whereas patients with cardiac arrest and digestive admissions were significantly higher in the non-SDD group (*p* < 0.05). The analysis, significantly, also reflects that there are more patients with ARDS in the non-SDD group than in the SDD group (*p* < 0.05). There were significantly more patients with one or more infections with carbapenemase-producing Gram-negative bacteria (GNB) and other multiresistant Gram-negative bacteria (MR GNB) in the non-SDD group compared to the SDD group (*p* < 0.05). We did not find statistically significant differences between the groups according to their AKI grade using the KDIGO scale.

Table 2 shows the nosocomial infection densities by SDD group, after excluding a statistically non-significant year, (*p* = 0.059) and the overdispersion effects (*p* = 0.925). In the models that were considered, the year did not show statistical significance, indicating stability among the infections. Likewise, none of the Poisson models that were considered showed statistically significant overdispersion. For nosocomial pneumonia, it can be seen that the rate decreased significantly, from 16.3 to 3.7 per one thousand days of mechanical ventilation (IDR: 0.17 (0.06–0.44)). For urinary infections, it can be seen that the rate decreased significantly, from 6.06 to 0.47 per one thousand days with a urinary catheter (IDR: 0.075 (0.009–0.64)). For catheter-related bacteremia (CRB), it can be seen that the rate decreased, but not significantly, from 2.65 to 0.57 per one thousand days with a catheter (IDR: 0.2 (0.019–2.254)). Concerning secondary bacteremia, it can be seen that the rate decreased, but not significantly, from 6.42 to 2.18 per one thousand days in the ICU (IDR: 0.35 (0.102–1.247)). Finally, concerning MDRB, it can be seen that the rate decreased significantly, from 15.42 to 2.33 per one thousand days in the ICU (IDR: 0.156 (0.048–0.5)).

**Figure 1:**
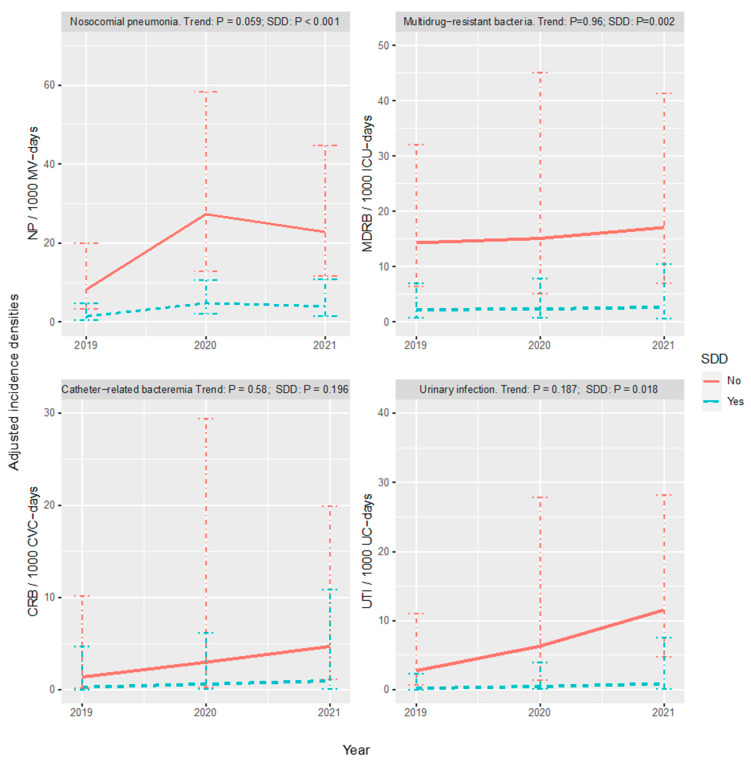
Evolution of incident density ratios according to SDD regime. Predictions of infection density incidences by year and SDD group, adjusted by Poisson models. Horizontal lines represent the 95% CI (confidence intervals). No infection type showed a statistically significant trend in incidence density over the follow-up period. SDD usage was associated with a reduction in risk for all types of infections except for CRB. NP: nosocomial pneumonia; MV: mechanical ventilation; MDRB: multidrug-resistant bacteria; CRB: catheter-related bacteremia; CVC: central venous catheter; UTI: urinary tract infection; SDD; selective digestive decontamination.

Figure 1 represents the evolution of the incident density ratio according to SDD regime over the three years under study. No infection type showed a statistically significant trend in incidence densities over the follow-up period. SDD usage was associated with a reduction in risk for all types of infections except for CRB.

Table 3 summarizes the patients’ characteristics according to their CRRT infection groups. This table also shows that 45 patients undergoing CRRT had infections, which corresponded to 32.1% of the sample. In total, 14 (31.1%) of these patients received SDD, while the remaining 31 patients (68.8%) did not. Of the 81 patients in the SDD group, only 14 were diagnosed with an infection related to CRRT, which corresponded to 17.2% of the group and 10% of the overall sample. In the non-SDD group, the remaining 31 patients (out of 59) had CRRT-linked infections, which corresponded to 52.5% of the group and 22.1% of the overall sample.

A multivariate logistic regression analysis of the CRRT infections is shown in Table 4. According to the Bayesian Information Criteria (BIC), the factors independently and statistically significantly associated with the outcome were the SDD regimen (OR = 0.10; 95% CI = 0.04; 0.26), peripheral vascular disease (OR = 3.17; 95% CI = 1.33; 7.56), and mechanical ventilation for at least seven days (OR = 7.91; 95% CI = 2.54; 24.66).

**Figure 2:**
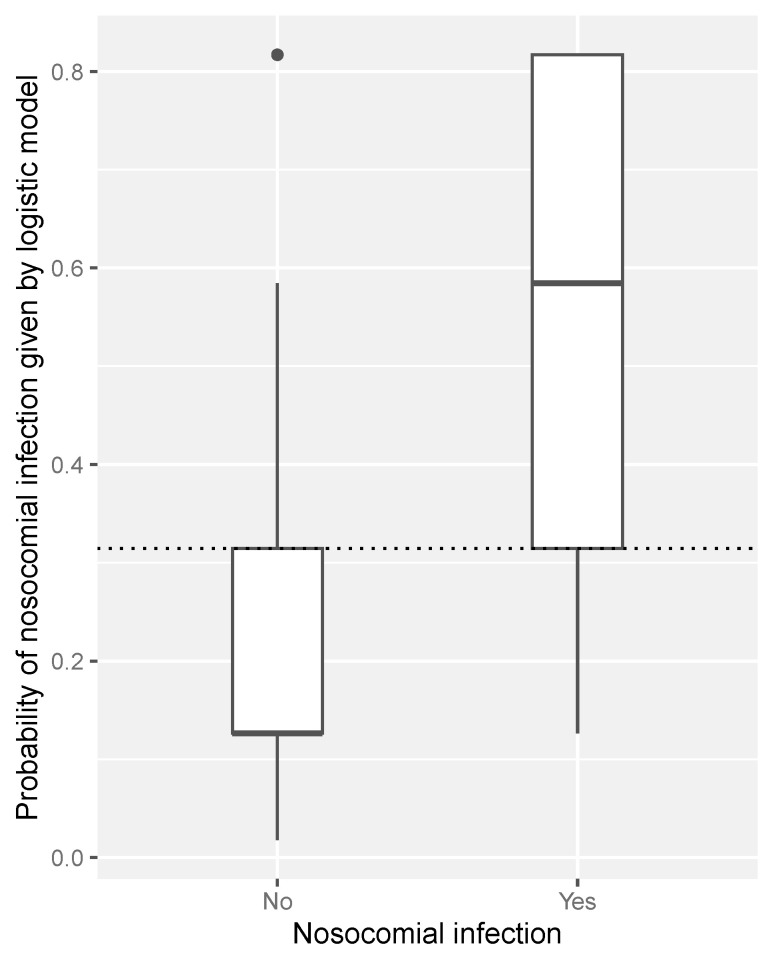
Boxplot of the adjusted probability of nosocomial infection according to the presence/absence of nosocomial infection. Note that, for the probability of infection, 0.315 is the 75th percentile among patients without infection and the 25th percentile among patients with infection.

Figure 2 displays a boxplot of the adjusted probability of nosocomial infection given by the logistic model according to the presence or absence of nosocomial infection. Thus, as shown in the figure, for 75% of patients without infection, the model assigns them a probability of infection of less than 31.5%, whereas only 25% of those with infection are assigned a probability of infection of less than 31.5%.

**Figure 3:**
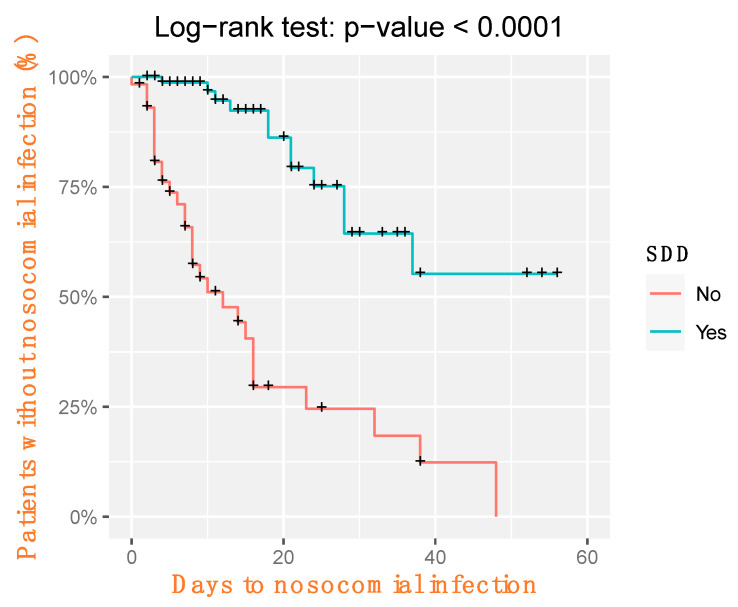
Survival time to nosocomial infection by SSD group. Survival curves corresponding the time from the start of CRRT to the incidence of infection were estimated by the Kaplan–Meier method and compared using the logrank test.

Figure 3 displays survival curves corresponding to the time from the start of CRRT to the incidence of infection, estimated by the Kaplan–Meier method and compared using the logrank test.

## 3. Discussion

In our study, we compared the incidences of ICU-acquired infections in two RRT cohorts at two tertiary hospitals in Spain, where the patients were exposed or not exposed to SDD. We demonstrated that SDD statistically significantly reduced RRT-associated nosocomial infections (OR: 0.10, 95% CI: (0.04–0.26)) and the MDRB incidence density ratio per one thousand days in the ICU (IDR: 0.156 (0.048–0.5)). In addition, seven or more days of mechanical ventilation (OR: 7.91, 95% CI: (2.54–24.66)) and peripheral vascular disease (OR: 3.17, 95% CI: (1.33–7.56)) were significantly associated with increases in nosocomial infections.

SDD has been shown to prevent serious infections [23,25] and may reduce mortality [26,27] in ICU patients. However, the use of this prophylactic tool is still controversial, particularly in ICUs with relatively high prevalences of MDR microorganisms [28,29], because it may contribute to or increase antimicrobial resistance [30]. In a previous study, we demonstrated that the long-term use of SDD was effective in reducing the rates of ventilator-associated pneumonia (VAP), secondary bloodstream infections, and antibiotic consumption while decreasing colistin-, tobramycin-, and antibiotic-resistant colonization rates in an ICU that applied SDD [13].

A recent meta-analysis published in *JAMA* in 2022 concluded that SDD decreases the risk of ventilator-associated pneumonia and in-hospital mortality [24]. These studies focused on general critically ill patients, and there are scarce references in the literature studying ICU subgroups, including patients with AKI requiring renal replacement therapy. Our results show that SDD protects against RRT-related nosocomial infections. More specifically, we observed in our study that, in an ICU environment where SDD was applied, the incidence density ratios of major nosocomial infections, such as ventilator-associated pneumonia, significantly decreased per one thousand days of mechanical ventilation (IDR: 0.17 (0.06–0.44)), highlighting the importance of adding SDD as a prophylactic treatment for nosocomial infections in patients with AKI on RRT.

We also sought to assess differences in the incidence of ICU-acquired secondary and MDR infections in critically ill RRT patients that were routinely placed in well-established ICUs in Spanish national infection control programs, according to the use of SDD. In our study, we found that SDD significantly decreased the incidence density ratio of infections caused by MDRB, which decreased from 15.42 to 2.33 per one thousand days in the ICU (IDR: 0.156 (0.048–0.5)). We acknowledge that there are many studies and guidelines that have highlighted RRT as a risk factor for nosocomial infections [4,5,31]. Moreover, the American IDSA guidelines even consider it a predisposing factor for the development of MDRB infections [5]. Regarding this, our results show the protective value of applying SDD to these patients that underwent RRT, as we previously showed in our setting with a mixed ICU population [13].

Our study also showed that SDD protected against the development of urinary tract infections in patients undergoing RRT in the ICU. In fact, it significantly decreased the urinary infection incidence density ratio per one thousand days with a urinary catheter (IDR: 0.075 (0.009–0.64)). There is controversy in the medical literature concerning the effects of SDD in reducing this incidence. Some authors have suggested that there may be translocation due to the proximity of the rectum and bladder, which may favor infection [32]. In this scenario, SDD could have a role in the prevention of urinary tract infections. On the other hand, other studies propose that the decrease in urinary tract infections is due to the entry of the prophylactic antibiotics of SDD into the bloodstream from the gastrointestinal tract, which could also favor the toxicity of these drugs [14,15,16]. We measured the plasma trough levels of tobramycin, gentamicin, and vancomycin in 52 patients in the SDD group, and we did not detect the presence of these drugs. Therefore, this favors translocation due to proximity as the pathogenesis of urinary infections, and in this setting SDD may influence the reduction in urinary tract infections. Our preliminary data also show that the SDD doses used were safe and did not produce secondary pharmacological toxicity.

Another finding of our study was decreases in bacteremia (both catheter-related and secondary to undetermined sources). For catheter-related bacteremia (CRB), we observed that the incidence density ratio non-significantly decreased from 2.65 to 0.57 per one thousand days with a catheter (IDR: 0.2 (0.019–2.254)). Concerning secondary bacteremia, it was observed that the rate decreased non-significantly from 6.42 to 2.18 per one thousand days in the ICU (IDR: 0.35 (0.102–1.247)). Several other studies in the literature have demonstrated an association between bacteremia and the gastrointestinal tract, either by direct translocation or the fecal contamination of surrounding catheters [31,33]. Falcone et al. demonstrated an increased risk of bloodstream infections from intestinal colonization by Klebsiella pneumoniae carbapenemases, specifically New Delhi metallo-β-lactamase (NDM) [34]. Therefore, in this scenario, SDD could contribute to decreases in colonization and bloodstream infections, as demonstrated by a Dutch group in 2011 [35].

The results obtained support the routine use of SDD as a prophylactic tool for nosocomial infection in ICU patients with renal failure and CRRT to improve infection control. Currently, more and more relevant guidelines and studies favor the implementation of SDD performed according to the original protocol, as carried out in early trials, as a recommended practice in ICUs. Otherwise, it simply will not work [36,37,38,39,40,41].

The main limitation of this study was its observational nature, as it was not a randomized clinical trial. It also had a small sample size, which may make it difficult to generalize the results. In addition, although the study compared two different patient groups and was performed in two nearby University Hospital ICUs on the same island that followed the same national ZERO infection control protocols [42] and that had previously worked together on a SDD study [43], there may be differences in clinical practice that may influence the study results.

In this study, 74 out of 140 patients died (52.85%), but we found no differences between the SDD and non-SDD cohorts in the hospitals or ICUs (*p* = 0.175). This was probably due to the small sample size, since there are studies in the literature showing that SDD reduces in-hospital mortality, such as a meta-analysis published in 2022 [24].

## 4. Materials and Methods

### 4.1. Study Design and Patients

An observational, multicenter, prospective cohort study was conducted at two tertiary acute care hospitals in Las Palmas de Gran Canaria, Canary Islands, Spain. This study included one hundred and forty consecutive critically ill patients, admitted to both ICUs between 1 January 2019 and 31 December 2021, who received CRRT for more than 48 h. They were grouped into two cohorts based on whether they received an SDD regimen. Patients recruited from one of the participating hospitals were included in the SDD cohort, and patients from the other hospital were included in the non-SDD cohort.

The inclusion criteria were as follows:

1. Age of >18 years.
2. Required RRT.
3. RRT duration of >48 h.

The exclusion criteria were as follows:

1. Sepsis or septic shock at ICU admission.
2. Duration of RRT of ≤48 h.

### 4.2. Study Variables

#### 4.2.1. Patients’ General Characteristics

The following characteristics were evaluated:

1. Age and sex.
2. Body mass index (BMI), in kg/m^2^.
3. Severity scales: Apache II score [44] and SOFA score [45].
4. Comorbidity scale: Charlson Comorbidity Index [46].
5. Patient comorbidities: COVID-19, heart failure, chronic renal failure, COPD, peripheral vascular disease, diabetes, dyslipidemia, and hypertension.
6. History of medications prescribed/used: Angiotensin-converting enzyme inhibitors (ACEIs), Angiotensin II receptor antagonists (ARA IIs), immunosuppressants, diuretics, metformin, and corticosteroids. We also recorded whether the patient had consumed antibiotics, undergone surgery, or been admitted to hospital in the previous 30 days, and we assessed whether the patient had a previous admission to an ICU.

#### 4.2.2. Other Variables

The following variables were evaluated:

1. Admission diagnosis: cardiac surgery, coronary artery disease, cardiac arrest, acute respiratory failure, and digestive pathology on admission to the ICU.
2. Shock on admission to the ICU and its type: cardiogenic, obstructive, hemorrhagic, and others.
3. Risk factors for nosocomial infections: neutropenia, surgery, corticosteroid use, acute respiratory distress syndrome (ARDS), ≥7 days of mechanical ventilation, parenteral nutrition, ≥5 days of central venous catheter use, and urinary catheter use. Renal failure variables: oliguric or non-oliguric renal failure and recovery.
4. Infections: The following nosocomial infections were diagnosed according to the ENVIN-HELICS criteria and recorded—nosocomial pneumonia, urinary tract infections, catheter-related bacteremia, and secondary bacteremia. The presence of the following infections caused by multidrug-resistant bacteria was recorded—methicillin-resistant Staphylococcus aureus (MRSA), vancomycin-resistant (VR) Enterococci, multiresistant (MR) Pseudomonas aeruginosa, carbapenemase-producing GNB, MDR GNB, and extended-spectrum β-Lactamases (ESBL) Enterobacteriaceae.

### 4.3. SDD Protocol

A well-established full SDD regimen commonly used in clinical practice in Spain was applied [13]. To receive SDD, patients must meet one or more of the following criteria:

- Orotracheal intubation expected for more than 48 h;
- Decreased level of consciousness;
- Burns;
- Neutropenia and/or transplant;
- Previous colonization by an MDRB;
- Severe pancreatitis.

This regimen was administered 3 times daily in patients that were mechanically ventilated for more than 48 h, starting on the day of orotracheal intubation and continuing until the day of discharge from the ICU. This regimen consisted of three components [13]: (1) 1 g of an oral paste applied in the oral cavity, composed of 20 mg of 2% colistin, 30 mg of 3% tobramycin, and 20 mg of 2% nystatin; (2) 14 mL of a suspension containing 140 mg of 1% colistin, 180 mg of 2% tobramycin, and 453.6 mg of 3.2% nystatin, which was administered into the intestine through a nasogastric tube; and (3) cefotaxime, 1 g every 8 h (or levofloxacin, 500 mg every 24 h, in the case of an allergy), which was administered during the first 4 days of treatment with SSD. In patients with methicillin-resistant *Staphylococcus aureus* (MRSA), a solution consisting of 40 mg of 4% oropharyngeal paste and 700 mg of vancomycin in a digestive solution was added to the above-mentioned regimen [13].

### 4.4. Definitions and Study Procedure

At ICU admission, and once a week, we collected surveillance samples from the rectum, throat, and airway, if the patient was endotracheally intubated. When a nosocomial infection was suspected, blood, urine, catheters, and respiratory microbiological samples were obtained. We used the ENVIN-HELICS diagnosis criteria for nosocomial infections [4], which were mainly catheter-related infections, ventilator-associated pneumonia (VAP), and urinary infections.

The preventable risk factors for the study endpoints were those established by the Spanish National Zero Infection Control Programs for ICU infection control [38,39,40,41].

### 4.5. Statistical Analysis

Design

This was a prospective study that included one hundred and forty critically ill patients.

Statistical analysis

Categorical variables are expressed as frequencies and percentages and continuous ones as means with standard deviation (SD) when data followed a normal distribution, or as medians and interquartile ranges (IQR = 25th–75th percentile) when their distribution departed from normality. The percentages were compared, as appropriate, using the Chi-square test or the exact Fisher test, the means by the t-test, and the medians by the Wilcoxon test for independent data. To identify the factors that maintained an independent association with continuous renal replacement therapy infections, a multivariate logistic regression analysis was performed. The variables that showed significant association with the outcome in the univariate analysis (*p* < 0.1) were entered into the multivariate analysis. A selection of variables, based on the best subset regression and Bayesian Information Criterion (BIC), was then performed. The model was summarized as *p*-values (likelihood ratio test) and odds ratios, which were estimated by means of confidence intervals at 95% [47].

Incidences per 1000 days of exposure. Infection rates were determined for each of the cohorts, defined by *SDD* regimen (Yes/No) and study year (2019, 2020, 2021). The exposure times for each cohort were obtained as the sum of the exposure times for all patients belonging to the cohort. Thus, for each one of k=2×3=6 cohorts described, their exposure days were obtained (mechanical ventilation, urinary catheter, central venous catheter, and ICU stay) and the incidences of the following infection types: nosocomial pneumonia, urine infection, catheter-related bacteremia, secondary bacteremia, and multiresistant germs. Then, for each infection type, we assume, according to Dean and Lawless (1989), that the number of events in the *k*-th cohort is a random variable *N_k_*~*Poisson*(*ν_k_* *μ_k_*):

ln(*μ_k_*) = ln(*days_k_*) + *α* + *β SDD_k_* + *γ*⋅*Year_k_*

Here, ln denotes the natural logarithm, *days_k_* the days of exposure of the entire cohort, *SDD_k_* = 1, 0 (presence or not of *SDD*), *Year_k_* denotes the year effect (2019 is taken as the reference and thus *γ*_2019_ = 0), and *ν*_1_ and *ν*_6_ are continuous positive-valued independent and identically distributed random variables of mean one and variance *τ* (overdispersion). The adjusted incidence density ratio (*SDD* versus no *SDD*) is obtained as exp(*β*). The model was estimated by the likelihood method and summarized by the incidence density ratio, which were estimated by means confidence intervals at 95%.

Survival time to CRRT infection by *SSD* group. Survival curves corresponding to the time from the start of CRRT to the incidence of infection were estimated by the Kaplan–Meier method and compared using the logrank test.

Statistical significance was set at *p* < 0.05. Data were analyzed using the R package, version 4.2.1 (R Development Core Team, 2022) [48].

## 5. Conclusions

In a multicenter, prospective, observational cohort study at two tertiary hospitals that compared the incidences of ICU-acquired infections, including MDRB, in two RRT cohorts where patients were exposed or not exposed to SDD, the SDD cohort had a statistically significantly reduced rate of RRT-associated nosocomial infections, showing that SDD may protect against RRT-related nosocomial infections. It was shown that the MDRB incidence density ratio significantly decreased per one thousand days in the ICU. The results obtained favor the use of SDD in ICU patients with renal failure undergoing CRRT as a tool for infection control.

## Figures and Tables

**Table 1:** Demographic data and patient characteristics.

	Overall N = 140	Non-SDD N = 59	SDD N = 81	*p*-Value
Age (years)	61.4 ± 14.7	58.9 ± 15.1	63.2 ± 14.2	0.087
Sex (female), n (%)	45 (32.1)	19 (32.2)	26 (32.1)	0.99
BMI (kg/m^2^), mean ± SD	29.0 ± 5.9	29.1 ± 6.4	28.9 ± 5.5	0.865
APACHE II	21.0 ± 6.6	18.3 ± 6.8	23.0 ± 5.8	<0.001
SOFA	8.1 ± 3.1	6.9 ± 2.7	8.9 ± 3.2	<0.001
Death, n (%)				0.175
No	66 (47.1)	23 (39.0)	43 (53.1)	
ICU	66 (47.1)	31 (52.5)	35 (43.2)	
Hospital	8 (5.7)	5 (8.5)	3 (3.7)	
ICU stay, median (IQR) days	21 (10; 39.2)	17 (8; 38.5)	23 (12; 39)	0.066
Mechanical ventilation days, median (IQR)	17 (7.5; 32)	17 (6; 26.2)	18 (8; 35)	0.19
Charlson Comorbidity Index	4.8 ± 3.0	4.3 ± 2.7	5.2 ± 3.1	0.081
COVID-19, n (%)	21 (15.0)	10 (16.9)	11 (13.6)	0.581
Liver disease, n (%)	9 (6.4)	2 (3.4)	7 (8.6)	0.303
Cardiac failure, n (%)	40 (28.6)	9 (15.2)	31 (38.3)	0.003
Chronic renal failure, n (%)	41 (29.3)	14 (23.7)	27 (33.3)	0.218
COPD, n (%)	19 (13.6)	9 (15.2)	10 (12.3)	0.62
Peripheral vascular disease, n (%)	63 (45.0)	27 (45.8)	36 (44.4)	0.877
Diabetes, n (%)	62 (44.3)	27 (45.8)	35 (43.2)	0.764
Dyslipidemia, n (%)	80 (57.1)	30 (50.9)	50 (61.7)	0.199
Hypertension, n (%)	98 (70.0)	34 (57.6)	64 (79.0)	0.006
ACEIs, n (%)	39 (27.9)	17 (28.8)	22 (27.2)	0.829
ARA II, n (%)	31 (22.1)	4 (6.8)	27 (33.3)	<0.001
Immunosuppressants, n (%)	13 (9.3)	4 (6.8)	9 (11.1)	0.383
Diuretics, n (%)	46 (32.9)	15 (25.4)	31 (38.3)	0.11
ATBs in previous 30 d, n (%)	41 (29.3)	16 (27.1)	25 (30.9)	0.631
Surgery in previous 30 d, n (%)	25 (17.9)	8 (13.6)	17 (21.0)	0.257
Patients with previous ICU admission, n (%)	24 (17.1)	8 (13.6)	16 (19.8)	0.337
Corticosteroids, n (%)	18 (12.9)	7 (11.9)	11 (13.6)	0.765
Hospital admission in previous 30 d, n (%)	76 (54.3)	28 (47.5)	48 (59.3)	0.166
ICU admission diagnosis, n (%)				
Cardiac surgery	33 (23.6)	0	33 (40.7)	<0.001
Coronary artery disease	18 (12.9)	4 (6.8)	14 (17.3)	0.067
Cardiac arrest	13 (9.3)	9 (15.2)	4 (4.9)	0.038
Acute respiratory failure	61 (43.6)	29 (49.1)	32 (39.5)	0.256
Digestive	12 (8.6)	9 (15.2)	3 (3.7)	0.016
Shock, n (%)	59 (42.1)	25 (42.4)	34 (42.0)	0.962
Cardiogenic	32 (22.9)	5 (8.5)	27 (33.3)	<0.001
Obstructive	3 (2.1)	1 (1.7)	2 (2.5)	1
Hemorrhagic	11 (7.9)	7 (11.9)	4 (4.9)	0.202
Others	15 (10.7)	12 (20.3)	3 (3.7)	0.002
MOF, n (%)	54 (38.6)	15 (25.4)	39 (48.1)	0.006
Previous ICU admission for RF, n (%)	106 (76.8)	47 (79.7)	59 (74.7)	0.493
RF in ICU, n (%)	34 (24.6)	13 (22.0)	21 (26.6)	0.54
Acute renal failure, n (%)	102 (73.9)	44 (74.6)	58 (73.4)	0.878
Oliguric RF, n (%)	129 (93.5)	56 (94.9)	73 (92.4)	0.732
Renal failure recovery, n (%)	29 (21.0)	14 (23.7)	15 (19.0)	0.499
Infections related to CRRT, n (%)	45 (32.1)	31 (52.5)	14 (17.3)	<0.001
Iodine contrast, n (%)	114 (81.4)	50 (84.8)	64 (79.0)	0.389
ICU neutropenia, n (%)	10 (7.1)	5 (8.5)	5 (6.2)	0.743
ICU surgery, n (%)	35 (25.0)	14 (23.7)	21 (25.9)	0.767
ICU corticosteroids, n (%)	114 (81.4)	51 (86.4)	63 (77.8)	0.193
ARDS, n (%)	60 (42.9)	32 (54.2)	28 (34.6)	0.02
≥7 days of MV, n (%)	100 (71.4)	37 (62.7)	63 (77.8)	0.051
Parenteral nutrition, n (%)	13 (9.3)	8 (13.6)	5 (6.2)	0.137
≥5 days with CVC, n (%)	130 (92.9)	50 (84.8)	80 (98.8)	0.002
≥5 days with UC, n (%)	124 (88.6)	49 (83.0)	75 (92.6)	0.08
MRSA, n (%)	3 (2.1)	1 (1.7)	2 (2.5)	1
VR *enterococcus*, n (%)	0	0	0	1
MR *Pseudomonas aeruginosa*, n (%)	16 (11.4)	6 (10.2)	10 (12.3)	0.689
ESBL Enterobacteriaceae, n (%)	36 (25.7)	14 (23.7)	22 (27.2)	0.646
Carbapenemase-producing GNB, n (%)	10 (7.1)	8 (13.6)	2 (2.5)	0.018
MDR GNB, n (%)	16 (11.4)	12 (20.3)	4 (4.9)	0.005
Diabetes, n (%)				0.274
No	79 (56.4)	33 (55.9)	46 (56.8)	
Type 1	7 (5.0)	5 (8.5)	2 (2.5)	
Type 2	54 (38.6)	21 (35.6)	33 (40.7)	

BMI: body mass index; SD: standard deviation; SDD: selective digestive decontamination; COPD: Chronic Obstructive Pulmonary Disease; ACEIs: Angiotensin-converting enzyme inhibitors; ARA II: Angiotensin II receptor antagonists; ATBs: antibiotics; D: days; ICU: intensive care unit; MOF: multiple organ failure; RF: renal failure; CRRT: continuous renal replacement therapy; ARDS: acute distress respiratory syndrome; MV: mechanical ventilation; CVC: central venous catheter; UC: urinary catheter; MRSA: methicillin-resistant Staphylococcus aureus; VR: Vancomycin-resistant; MDR: multidrug-resistant; ESBL: extended-spectrum beta-lactamase; GNB: Gram-negative bacteria.

**Table 2:** Nosocomial infections: events per 1000 days of exposure.

	No SDD	SDD	Incidence Density Ratios (95% CI) †	Year Effect (*p* ‡)	Overdispersion (*p*)
**Nosocomial pneumonia**	17	7	0.174 (0.069–0.443)	0.059	0.925
Days of mechanical ventilation	1039	1889			
Events per 1000 days	16.362	3.706			
**MDRB**	12	4	0.156 (0.048–0.506)	0.957	0.898
Days in ICU	778	1713			
Events per 1000 days	15.424	2.335			
**CRB**	3	1	0.206 (0.019–2.254)	0.578	0.868
Days with catheter	1129	1741			
Events per 1000 days	2.657	0.574			
**Secondary bacteremia**	5	5	0.356 (0.102–1.247)	0.061	0.901
Days in ICU	778	1713			
Events per 1000 days	6.427	2.919			
**Urinary infection**	8	1	0.075 (0.009–0.64)	0.187	0.867
Days with urinary catheter	1320	2097			
Events per 1000 days	6.061	0.477			

(†) Adjusted by year; (‡) Likelihood ratio test. SDD: selective digestive decontamination; BIC: Bayesian Information Criterion; CRB: catheter-related bacteremia; ICU: intensive care unit; MDRB: multidrug-resistant bacteria.

**Table 3:** Patient characteristics by CRRT infection.

		CRRT Infection		
	Overall N = 140	No N = 95	Yes N = 45	*p*-Value
Age (years)	61.4 ± 14.7	61.6 ± 15.3	60.8 ± 13.6	0.764
Sex (female), n (%)	45 (32.1)	32 (33.7)	13 (28.9)	0.57
BMI (kg/m^2^), mean ± SD	29.0 ± 5.9	28.8 ± 5.9	29.2 ± 5.9	0.75
APACHE II	21.0 ± 6.6	21.6 ± 6.4	19.7 ± 6.9	0.114
SOFA	8.1 ± 3.1	8.2 ± 3.2	7.8 ± 3.0	0.416
Charlson Comorbidity Index	4.8 ± 3.0	4.5 ± 3.1	5.5 ± 2.6	0.084
SDD, n (%)	81 (57.9)	67 (70.5)	14 (31.1)	<0.001
Death, n (%)				0.228
No	66 (47.1)	49 (51.6)	17 (37.8)	
ICU	66 (47.1)	42 (44.2)	24 (53.3)	
Hospital	8 (5.7)	4 (4.2)	4 (8.9)	
ICU stay, median (IQR)	21 (10; 39.2)	17 (9; 32.5)	31 (17; 50)	0.002
MV days, median (IQR)	17 (7.5; 32)	13.5 (5; 28.8)	24 (17; 40)	0.002
COVID-19, n (%)	21 (15.0)	13 (13.7)	8 (17.8)	0.526
Cardiac failure, n (%)	40 (28.6)	26 (27.4)	14 (31.1)	0.647
Chronic renal failure, n (%)	41 (29.3)	30 (31.6)	11 (24.4)	0.386
COPD, n (%)	19 (13.6)	8 (8.4)	11 (24.4)	0.01
Peripheral vascular disease, n (%)	63 (45.0)	36 (37.9)	27 (60.0)	0.014
Diabetes, n (%)	62 (44.3)	40 (42.1)	22 (48.9)	0.45
Dyslipidemia, n (%)	80 (57.1)	51 (53.7)	29 (64.4)	0.23
Hypertension, n (%)	98 (70.0)	63 (66.3)	35 (77.8)	0.167
ACEIs, n (%)	39 (27.9)	21 (22.1)	18 (40.0)	0.027
ARA II, n (%)	31 (22.1)	24 (25.3)	7 (15.6)	0.196
Immunosuppressants, n (%)	13 (9.3)	8 (8.4)	5 (11.1)	0.756
Diuretics, n (%)	46 (32.9)	32 (33.7)	14 (31.1)	0.762
Metformin, n (%)	28 (20.0)	17 (17.9)	11 (24.4)	0.366
ATBs in previous 30 d, n (%)	41 (29.3)	27 (28.4)	14 (31.1)	0.744
Surgery in previous 30 d, n (%)	25 (17.9)	21 (22.1)	4 (8.9)	0.057
Previous ICU admission, n (%)	24 (17.1)	17 (17.9)	7 (15.6)	0.732
Corticosteroids, n (%)	18 (12.9)	12 (12.6)	6 (13.3)	0.908
Hospital admission in previous 30 d, n (%)	76 (54.3)	52 (54.7)	24 (53.3)	0.876
**ICU admission diagnosis, n (%)**				
Cardiac surgery	33 (23.6)	27 (28.4)	6 (13.3)	0.049
Coronary artery disease	18 (12.9)	14 (14.7)	4 (8.9)	0.334
Cardiac arrest	13 (9.3)	10 (10.5)	3 (6.7)	0.549
Acute respiratory failure	61 (43.6)	38 (40.0)	23 (51.1)	0.216
Digestive	12 (8.6)	8 (8.4)	4 (8.9)	1
**Shock, n (%)**	59 (42.1)	39 (41.0)	20 (44.4)	0.704
Cardiogenic	32 (22.9)	23 (24.2)	9 (20.0)	0.58
Obstructive	3 (2.1)	3 (3.2)	0	0.551
Hemorrhagic	11 (7.9)	9 (9.5)	2 (4.4)	0.503
Others	15 (10.7)	5 (5.3)	10 (22.2)	0.006
MOF, n (%)	54 (38.6)	34 (35.8)	20 (44.4)	0.326
Previous ICU admission for RF, n (%)	106 (76.8)	69 (74.2)	37 (82.2)	0.295
RF during ICU stay, n (%)	34 (24.6)	25 (26.9)	9 (20.0)	0.379
Acute renal failure, n (%)	102 (73.9)	68 (73.1)	34 (75.6)	0.76
Oliguric RF, n (%)	129 (93.5)	87 (93.5)	42 (93.3)	1
Renal failure recovery, n (%)	29 (21.0)	21 (22.6)	8 (17.8)	0.516
Iodine contrast, n (%)	114 (81.4)	70 (73.7)	44 (97.8)	<0.001
ICU neutropenia, n (%)	10 (7.1)	6 (6.3)	4 (8.9)	0.727
ICU surgery, n (%)	35 (25.0)	20 (21.1)	15 (33.3)	0.117
ICU corticosteroids, n (%)	114 (81.4)	72 (75.8)	42 (93.3)	0.013
ARDS, n (%)	60 (42.9)	32 (33.7)	28 (62.2)	0.001
≥7 days of MV, n (%)	100 (71.4)	61 (64.2)	39 (86.7)	0.006
Parenteral nutrition, n (%)	13 (9.3)	6 (6.3)	7 (15.6)	0.116
≥5 days with CVC, n (%)	130 (92.9)	86 (90.5)	44 (97.8)	0.168
≥5 days with UC, n (%)	124 (88.6)	80 (84.2)	44 (97.8)	0.018
MRSA, n (%)	3 (2.1)	3 (3.2)	0	0.551
VR *enterococcus*, n (%)	0	0	0	1
MR *Pseudomonas aeruginosa*, n (%)	16 (11.4)	8 (8.4)	8 (17.8)	0.104
ESBL Enterobacteriaceae, n (%)	36 (25.7)	20 (21.1)	16 (35.6)	0.067
Carbapenemase-producing GNB, n (%)	10 (7.1)	3 (3.2)	7 (15.6)	0.013
MDR GNB, n (%)	16 (11.4)	7 (7.4)	9 (20.0)	0.028

BMI: body mass index; SDD: selective digestive decontamination; COPD: Chronic Obstructive Pulmonary Disease; ACEIs: Angiotensin-converting enzyme inhibitors; ARA II: Angiotensin II receptor antagonists; ATBs: antibiotics; D: days; ICU: intensive care unit; MOF: multiple organ failure; RF: renal failure; CRRT: continuous renal replacement therapy; ARDS: acute distress respiratory syndrome; MV: mechanical ventilation; CVC: central venous catheter; UC: urinary catheter; MRSA: methicillin-resistant Staphylococcus aureus; VR: Vancomycin-resistant; MDR: multidrug-resistant; ESBL: extended-spectrum beta-lactamase; GNB: Gram-negative bacteria.

**Table 4:** Multivariate logistic regression of CRRT infections.

	*p*-Value (†)	BIC (‡)	Odds Ratio (95% CI)
SDD	<0.001	176.5	0.10 (0.04; 0.26)
Peripheral vascular disease	0.007	155.5	3.17 (1.33; 7.56)
≥7 days of MV	<0.001	164.3	7.91 (2.54; 24.66)

BIC: Bayesian Information Criteria; SDD: selective digestive decontamination; MV: mechanical ventilation. †: likelihood ratio test. ‡: The BIC if this factor is removed. The BIC is a measure of a lack of fit. For the full model, BIC = 153.2. Note that if any of the factors are removed, the BIC value is increased, worsening the fit.

## Data Availability

Please contact the authors for data requests.
